# Behavioral Deficits in Juveniles Mediated by Maternal Stress Hormones in Mice

**DOI:** 10.1155/2016/2762518

**Published:** 2015-12-27

**Authors:** Jamie Maguire, Istvan Mody

**Affiliations:** ^1^Department of Neuroscience, Tufts University School of Medicine, 136 Harrison Avenue, Boston, MA 02111, USA; ^2^Departments of Neurology and Physiology, The David Geffen School of Medicine, University of California, 635 Charles Young Dr. South, Los Angeles, CA 90095, USA

## Abstract

Maternal depression has been shown to negatively impact offspring development. Investigation into the impact of maternal depression and offspring behavior has relied on correlative studies in humans. Further investigation into the underlying mechanisms has been hindered by the lack of useful animal models. We previously characterized a mouse model which exhibits depression-like behaviors restricted to the postpartum period and abnormal/fragmented maternal care (*Gabrd* ^−/−^ mice). Here we utilized this unique mouse model to investigate the mechanism(s) through which maternal depression-like behaviors adversely impact offspring development. Cross-fostering experiments reveal increased anxiety-like and depression-like behaviors in mice reared by *Gabrd* ^−/−^ mothers. Wild type and *Gabrd* ^−/−^ mice subjected to unpredictable stress during late pregnancy exhibit decreased pup survival and depression-like behavior in the postpartum period. Exogenous corticosterone treatment in wild type mice during late pregnancy is sufficient to decrease pup survival and induce anxiety-like and depression-like behaviors in the offspring. Further, the abnormal behaviors in juvenile mice reared by *Gabrd* ^−/−^ mice are alleviated by treatment of the mothers with the corticotropin-releasing hormone (CRH) antagonist, Antalarmin. These studies suggest that hyperresponsiveness of the HPA axis is associated with postpartum depression and may mediate the adverse effects of maternal depression on offspring behavior.

## 1. Introduction

Maternal depression has adverse effects on infant behavioral, emotional, and cognitive development [1–7]. Studies investigating the impact of postpartum depression on child development have largely relied on correlative studies in humans. However, investigation into the mechanisms mediating the transmission of negative affect from depressed mother to child has been impeded by the lack of useful animal models. We previously characterized a mouse model with deficits in maternal care which exhibits depression-like behaviors during the postpartum period (*Gabrd* ^−/−^ mice) [8]. Here we utilize this mouse model to investigate the mechanisms underlying the negative impact of maternal depression-like behaviors on offspring development.

Deficits in offspring development associated with maternal depression are correlated with elevated levels of stress hormones in both the mother and the fetus [9]. Treatment of pregnant women with exogenous glucocorticoids results in deficits in child development similar to those related to postpartum depression [10], suggesting that the stress response may mediate the adverse effects of maternal depression on offspring development (for review see [11]). The body's physiological response to stress is mediated by the hypothalamic-pituitary-adrenal (HPA) axis and involves the release of CRH from the hypothalamus, which triggers the release of adrenocorticotropic hormone (ACTH) from the pituitary, ultimately resulting in glucocorticoid (corticosterone in mice and cortisol in humans) release from the adrenal gland. Although basal levels of corticosterone increase throughout pregnancy and lactation, stress-induced elevations in stress hormones are suppressed (for review see [12]), which is thought to protect the fetus from the negative effects of exposure to high levels of glucocorticoids [13, 14]. Accumulating evidence suggests that dysregulation of the HPA axis plays a role in postpartum depression. The HPA axis may be hyperresponsive in postpartum depression, as indicated by elevated levels of cortisol [15], although these findings are controversial [16, 17]. More convincing evidence exists for increased levels of CRH [18, 19] and ACTH [18] associated with postpartum depression. Accordingly, it has been suggested that CRH levels are increased in women with postpartum depression and may even be used as a diagnostic criteria for postpartum depression [19]. Elevated levels of stress hormones in the depressed mother result in elevated levels of stress hormones in the infant [20] and higher levels of stress hormones are associated with decreased maternal care and offspring anxiety [21]. Therefore, it is reasonable to hypothesize that dysregulation of the HPA axis may play a role not only in postpartum depression but also in the negative impact of maternal depression on offspring development. Consistent with this hypothesis administration of exogenous corticosterone to the dams during the postpartum period induced behavioral abnormalities in the offspring [22].

The activity of the HPA axis is governed by CRH neurons in the paraventricular nucleus (PVN) of the hypothalamus (for review see [23]). The activity of these neurons, and thus activity of the HPA axis, is tightly regulated by robust GABAergic inhibition (for review see [24–26]), including tonic GABAergic inhibition mediated by *δ* subunit-containing GABA_A_Rs [27]. Interestingly, we demonstrated that a mouse model deficient in the GABA_A_R  *δ* subunit (*Gabrd* ^−/−^ mice) exhibit depression-like behaviors exclusively during the postpartum period and deficits in maternal behaviors [8]. A recent study confirmed these findings demonstrating that* Gabrd* ^−/−^ dams provide fragmented maternal care and their offspring exhibit phenotypes similar to those subjected to early life stress [28].

In this study, we utilized this mouse model (*Gabrd* ^−/−^ mice), which exhibit abnormal/fragmented maternal care and depression-like behavior during the postpartum period [8, 28], to investigate the impact of maternal depression-like behavior on offspring development and the involvement of stress-related steroid hormones. It is well known that maternal depression negatively impacts child development in humans [1–7]. Here we reproduce analogous deficits in offspring development associated with maternal depression-like behavior in* Gabrd* ^−/−^ dams which can be mimicked with unpredictable stress or exogenous corticosterone administration in wild type mice. Further, blocking CRH signaling with Antalarmin during pregnancy in* Gabrd* ^−/−^ mice prevents the adverse behavioral effects on the juvenile offspring. This study demonstrates the utility of* Gabrd* ^−/−^ mice in investigating the pathophysiological mechanisms of postpartum depression and implicates hyperexcitability of the HPA axis in postpartum depression-like behavior and the negative impact on offspring development.

## 2. Materials and Methods

### 2.1. Animal Handling and Treatment

Adult (3 months old) C57/Bl6 and* Gabrd* ^−/−^ mice were housed at the University of California, Los Angeles (UCLA), Division of Laboratory Animal Medicine. The animals (4/cage) were housed in clear plastic cages in a temperature- and humidity-controlled environment with a 12 h light/dark cycle (light on at 6 a.m.) and were maintained on an* ad libitum* diet of lab chow and water. Animals were handled according to protocols approved by the UCLA, Chancellor's Animal Research Committee (ARC). For the pregnancy experiments, C57/Bl6 and* Gabrd* ^−/−^ adult female mice were exposed to a male mouse for a single dark cycle. The female was checked for a vaginal plug and placed into a separated home cage. The pregnant female was individually housed with the single litter until the pups were weaned. For the cross-fostering experiments, the mothers were removed from the native litter and swapped with a surrogate (either wild type or* Gabrd* ^−/−^) immediately following delivery. The mothers were exchanged to minimize the disturbance and handling of the pups. The juvenile offspring remained in the home cage with the mother (or surrogate) and littermates until all behavioral testing was complete.

### 2.2. Unpredictable Stress Paradigm

This study utilized a stress paradigm devised by another research team, which they termed “chronic ultramild stress” [29, 30] and has been shown to elevate corticosterone levels during pregnancy [29, 30]. In the current study, this stress paradigm is referred to as unpredictable stress (US). Wild type and* Gabrd* ^−/−^ mice were subjected to unpredictable stress from D14 to D21 of pregnancy as previously described [29, 30]. Wild type and* Gabrd* ^−/−^ mice at D14 of pregnancy were randomly assigned to two groups: Group 1 (stressed) were subjected to an unpredictable stressor (cage tilt, confinement in a small cage, overnight light exposure for a single night, soiled cage for a single 24-hour period, and difficult access to food) during the dark period for 7 consecutive days until D21 of pregnancy unless parturition occurred before at which time subjection to the stressors was immediately ceased. The stressors were alternated to prevent habituation to a single stressor. The periods of stress were separated by stress-free intervals of at least 12 hours. Group 2 (controls) were maintained in their home cage without subjection to the unpredictable stressors.

### 2.3. Behavioral Tests

Behavioral tests were performed on wild type and* Gabrd* ^−/−^ dams at 48 hrs postpartum, a time point which has previously been demonstrated to be associated with abnormal postpartum and maternal behaviors in* Gabrd* ^−/−^ mice [8]. Behavioral tests in juvenile mice began at P21 with the open field test followed by the more stressful forced swim test 24 hrs later. P21 was chosen as the time point to test the impact of maternal behavior on the behavior of the offspring since this time point is prior to weaning and at this time the offspring still share a home cage with the mother.

#### 2.3.1. Depression Assay: Porsolt Forced Swim Test

Depression-like behavior was assessed in wild type and* Gabrd* ^−/−^ dams subjected to unpredictable stress (US) at 48 hrs postpartum and in cross-fostered, CORT, and Antalarmin-treated offspring at approximately P21 using the forced swim test as previously described [8, 31]. Briefly, each mouse was placed individually in a glass cylinder (21 cm × 12 cm), containing 9 cm of room temperature water (22–25°C), in which there is no escape. The latency to immobility and the total duration of immobility throughout the 6 min forced swim test were measured. The mouse was considered to be immobile when it ceased swimming and remained floating motionless, except for infrequent movements of a single hindlimb to maintain being afloat. All tests were videotaped and subsequently analyzed and scored.

#### 2.3.2. Anxiety Assay: Open Field Test

Anxiety-like behavior was assessed in cross-fostered, CORT, and Antalarmin-treated juvenile offspring at P21 using the open field test. Animals were tested in the same testing area, under bright light, with no visual cues. The apparatus was cleaned with ethanol and water in between animals to prevent olfactory cues. The mice were placed into the center of the open field, which consists of a plexiglass container (40 × 30 × 40 cm) with a grid of squares (10 × 7) on the bottom. The amount of time spent in the center (6 × 3) squares was measured over a single 10 min testing period. The total number of lines crossed (beam breaks) during the 10 min test was also counted.

### 2.4. Acute Stress Paradigm

The acute stress paradigm was utilized to measure stress-induced elevations in corticosterone in virgin and postpartum wild type and* Gabrd* ^−/−^ mice. An adapted protocol of the CO_2_ exposure paradigm [32] in adult female wild type and* Gabrd* ^−/−^ mice was used as an acute stressor. Exposure to 35% CO_2_ for 2 min was used to produce marked increases in circulating corticosterone levels [33]. Virgin and postpartum (48 hrs) wild type and* Gabrd* ^−/−^ mice were randomly assigned to two groups: Group 1 (stressed) which were subjected to a single episode of CO_2_ stress (35% for 2 min) and Group 2 (controls) which were handled in a similar way as Group 1 except they received air instead of air enriched with CO_2_. All animals were handled similarly in which their home cage was inserted into a larger ventilation box where CO_2_ (or air in the case of controls) was administered. The animals were allowed to recover for 30 min prior to blood collection.

### 2.5. Steroid Hormone Concentration Determination

Blood was collected for corticosterone measurements from wild type and* Gabrd* ^−/−^ mice 30 min following acute CO_2_ stress and compared to controls. Mice were anesthetized with isoflurane before whole blood was collected from experimental groups by retro-orbital bleeding between 12 and 14 hrs. Plasma was immediately isolated by high speed centrifugation and stored at −20° until use. Corticosterone levels were measured by enzyme immunoassay according to manufacturer's specifications (Enzo Life Sciences) as described previously [27, 34–36]. Briefly, triplicate 5 *μ*L plasma samples were assayed and compared to a standard curve using a spectrophotometer (at 450 nm). Intra-assay variability of the corticosterone assay was 7.8 ng/mL between paired samples and the interassay variability was 4.4 ng/mL for the same samples between assays.

### 2.6. Corticosterone Implantation

Wild type mice at day 14 (D14) of pregnancy were briefly anaesthetized with halothane until unresponsive to a foot pinch and were either sham implanted or implanted with a 21-day release 10 mg corticosterone pellet (Innovative Research of America, Sarasota, FL). The hair from the incision site on the back of the neck was clipped and swabbed with ethanol and iodine prior to making the incision. A small 1 cm incision was made on the back of the neck and a small slow-release pellet (or nothing for sham) was placed underneath the skin using forceps without touching the external area. The incision was then closed with sutures. Pup survival was determined at postnatal day 7 (P7) and offspring behavior was assessed in the juveniles at P21. Two mice per litter were randomly selected for behavioral analysis.

### 2.7. Antalarmin Treatment

Antalarmin was administered to* Gabrd* ^−/−^ mice from D14 to D21 of pregnancy in the drinking water to minimize the handling of the animals. At day 14 of pregnancy, the normal drinking water was replaced with the Antalarmin solution (10 mg Antalarmin/10 *μ*L ethanol/100 mL drinking water). The animals were maintained on either vehicle (10 *μ*L ethanol/100 mL drinking water) or Antalarmin until D21 of pregnancy or immediately after parturition (if before D21) at which time the animals were returned to normal drinking water. This treatment strategy was previously shown to block elevations in corticosterone levels [35, 36]. Pup survival was assessed at P7 and offspring behavior was assessed at P21.

### 2.8. Statistics

A one-way ANOVA with Tukey's post hoc multiple comparisons test was used to determine statistical significance for comparing more than two experimental groups. Student's *t*-test was used to determine statistical significance between two experimental groups. All statistical tests were carried out using Graphpad 6.0 (Prism).

## 3. Results

### 3.1. Impact of Maternal Depression-Like Behaviors in* Gabrd* ^−/−^ Mice on Offspring Behavior

**Figure 1:**
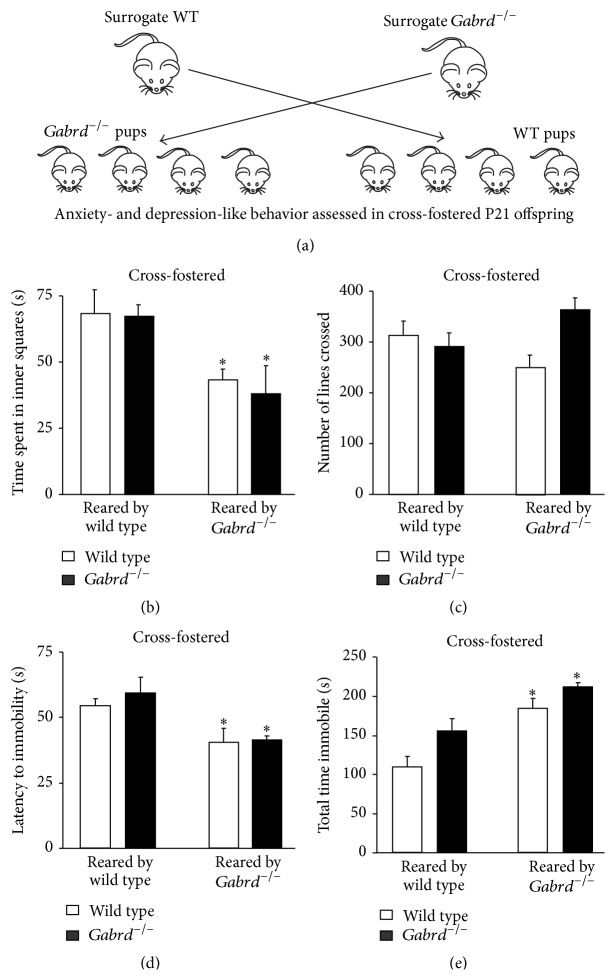
Maternal depression-like behaviors negatively impact offspring behavior. (a) A diagram outlining the experimental design for the cross-fostering experiments. Wild type and* Gabrd* ^−/−^ mice were reared by either a surrogate wild type or surrogate* Gabrd* ^−/−^ mother. Juvenile wild type and* Gabrd* ^−/−^ offspring reared by surrogate mothers exhibiting depression-like behavior (*Gabrd* ^−/−^ mice) exhibit anxiety-like behavior in the open field test, evident by a significant decrease in the time spent in the center squares (b) with no change in the number of lines crossed (c) during the 10 min test. *n* = 12–17 mice per experimental group; *∗* denotes *p* < 0.05 using a one-way ANOVA with Tukey's multiple comparisons test. Juvenile wild type and* Gabrd* ^−/−^ offspring reared by surrogate mothers exhibiting depression-like behavior (*Gabrd* ^−/−^ mice) also exhibit depression-like behavior, assessed using the forced swim test. Offspring reared by surrogate* Gabrd* ^−/−^ mothers exhibit a significant decreased latency to immobility (d) and an increase in the total time spent immobile (e) compared to offspring reared by surrogate wild type mothers. *n* = 10–12 mice per experimental group; *∗* denotes *p* < 0.05 using one-way ANOVA with Tukey's multiple comparisons test.

Here we utilized* Gabrd* ^−/−^ mice [8] to investigate the impact of maternal depression-like behaviors on offspring behavior. To determine if the maternal behavior,* per se*, directly mediates the deficits in offspring behavior, we performed cross-fostering experiments. Immediately following delivery, the natural mothers of wild type or* Gabrd* ^−/−^ litters were replaced with a surrogate wild type or* Gabrd* ^−/−^ mother and the behavior of the cross-fostered animals was then assessed in the juvenile offspring at age P21 (Figure 1(a)). Our data demonstrate that juvenile wild type or* Gabrd* ^−/−^ mice reared by mice exhibiting depression-like behavior during the postpartum period (*Gabrd* ^−/−^ mice) exhibit increased anxiety-like behavior in the open field test compared to juvenile wild type or* Gabrd* ^−/−^ mice reared by surrogate wild type mothers (Table 1; Figure 1(b)). Juvenile mice reared by* Gabrd* ^−/−^ mice spent less time in the center squares of the open field test compared to juvenile mice reared by wild type mothers (Table 1; Figure 1(b)) (*n* = 12–17 mice per experimental group; *∗* denotes *p* < 0.05 using a one-way ANOVA with Tukey's multiple comparisons test; *F*(3,54) = 7.778). However, there is no significant difference in the locomotor behavior, indicated by the number of lines crossed, between offspring reared by wild type and* Gabrd* ^−/−^ mothers (Table 1; Figure 1(c)) (*n* = 12–17 mice per experimental group; *∗* denotes *p* < 0.05 using a one-way ANOVA with Tukey's multiple comparisons test; *F*(3,54) = 2.819). These results suggest that maternal depression-like behaviors negatively impact offspring development, resulting in increased anxiety-like behavior. In addition, both wild type and* Gabrd* ^−/−^ juvenile mice, reared by* Gabrd* ^−/−^ mothers, exhibit an increase in depression-like behavior compared to mice reared by surrogate wild type mothers. Juvenile mice reared by* Gabrd* ^−/−^ mice exhibit a decreased latency to immobility and an increase in the total time spent immobile in the forced swim test compared to juvenile mice reared by wild type mothers (Table 1; Figures 1(d) and 1(e)) (*n* = 10–12 mice per experimental group; *∗* denotes *p* < 0.05 using a one-way ANOVA with Tukey's multiple comparisons test; latency: *F*(3,39) = 4.386; total time: *F*(3,39) = 12.61). These data demonstrate that maternal depression-like behaviors in* Gabrd* ^−/−^ mice during the postpartum period negatively impact offspring development and validate the use of this model for investigating the mechanisms mediating the negative impact of maternal depression on offspring development.

### 3.2. Role of Stress in Abnormal Maternal and Postpartum Behaviors

**Figure 2:**
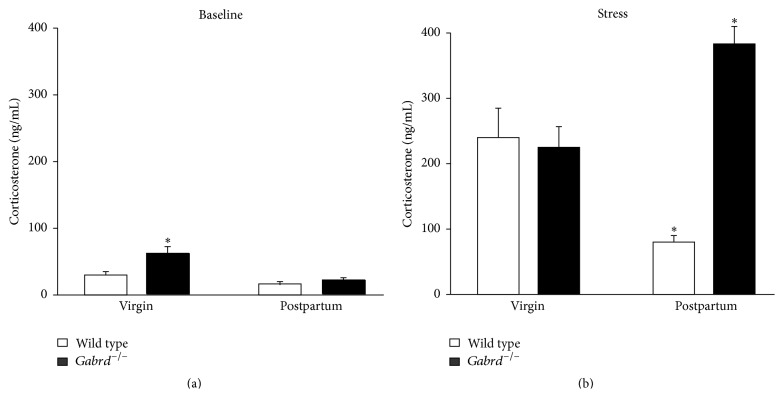
HPA axis hyperexcitability in postpartum* Gabrd* ^−/−^ mice. (a) Baseline corticosterone levels measured in the plasma of virgin and postpartum wild type and* Gabrd* ^−/−^ mice. (b) Average circulating corticosterone levels following CO_2_ stress in virgin and postpartum wild type and* Gabrd* ^−/−^ mice. (*n* = 7–17 mice per experimental group; *∗* denotes *p* < 0.05 using one-way ANOVA with Tukey's multiple comparisons test).

We proposed that the abnormal postpartum behaviors in* Gabrd* ^−/−^ mice may be associated with altered stress reactivity during the postpartum period. Postpartum* Gabrd* ^−/−^ mice exhibit an increase in corticosterone levels following acute CO_2_ stress (384.6 ± 27.6 ng/mL) compared to postpartum wild type mice (81.0 ± 10.4 ng/mL), virgin wild type mice (241.0 ± 45.3 ng/mL), or virgin* Gabrd* ^−/−^ mice (225.6 ± 32.7 ng/mL) (Figure 2). However, there is no significant difference in circulating corticosterone levels between unstressed postpartum* Gabrd* ^−/−^ mice (20.5 ± 4.5 ng/mL), postpartum wild type mice (16.8 ± 4.1 ng/mL), virgin* Gabrd* ^−/−^ mice (59.9 ± 10.6 ng/mL), or virgin wild type mice (30.7 ± 5.3 ng/mL) (Figure 2) (*n* = 7–17 mice per experimental group; *∗* denotes *p* < 0.05 using a one-way ANOVA with Tukey's multiple comparisons test; *F*(8,93) = 20.94).

**Figure 3:**
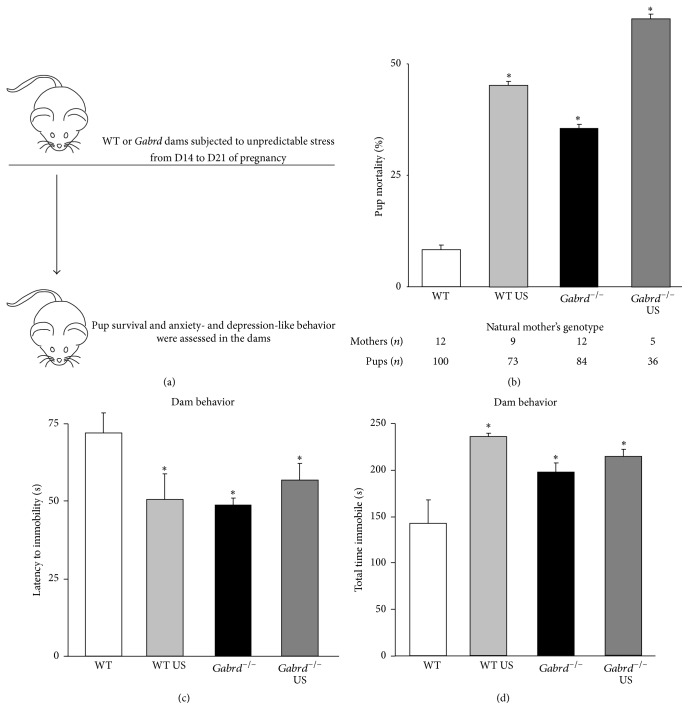
Unpredictable stress during pregnancy results in decreased pup survival and depression-like behavior in the dams. (a) A diagram outlining the experimental design. Wild type and* Gabrd* ^−/−^ dams were subjected to unpredictable stress from D14 to D21 of pregnancy and pup survival and depression-like behavior were assessed in the dams at 48 hrs postpartum. (b) Wild type and* Gabrd* ^−/−^ mice subjected to unpredictable stress from D14 to D21 of pregnancy exhibit a decrease in pup survival compared to control wild type and* Gabrd* ^−/−^ mice. *n*: wild type = 12 mothers, 100 pups; wild type US = 9 mothers, 73 pups;* Gabrd* ^−/−^ = 12 mothers, 84 pups;* Gabrd* ^−/−^ US = 5 mothers, 36 pups; *∗* denotes *p* < 0.05 using Student's *t*-test. Wild type and* Gabrd* ^−/−^ dams subjected to unpredictable stress from D14 to D21 of pregnancy exhibit a decreased latency to immobility (c) and an increased total time spent immobile (d) in the forced swim test at 48 hrs postpartum. *n* = 5–9 for each experimental group; *∗* denotes *p* < 0.05 using one-way ANOVA with Tukey's multiple comparisons test.

If altered stress reactivity plays a role in maternal depression-like behaviors in* Gabrd* ^−/−^ mice, then we hypothesized that chronic stress would be sufficient to induce the same behavioral disturbances in wild type mice during the postpartum period. Wild type mice subjected to unpredictable stress (US) from D14 to D21 of pregnancy (Figure 3(a)) exhibit a decrease in the survival rate of their pups compared to unstressed controls (Table 1; Figure 3(b)) (*n*: wild type = 12 mothers, 100 pups; wild type US = 9 mothers, 73 pups; *∗* denotes *p* < 0.05 using Student's *t*-test). Decreased pup survival is exacerbated in* Gabrd* ^−/−^ mice subjected to US compared to unstressed* Gabrd* ^−/−^ mice (Table 1; Figure 3(b)) (*n*:* Gabrd* ^−/−^ = 12 mothers, 84 pups;* Gabrd* ^−/−^ US: 5 mothers, 36 pups; *∗* denotes *p* < 0.05 using Student's *t*-test). Dams subjected to US also fail to build a nest and keep the pups at an increased distance from the mother (data not shown), similar to* Gabrd* ^−/−^ mice [8]. These abnormal maternal behaviors in wild type mice subjected to the US paradigm are associated with depression-like behaviors in the dams (Figures 3(c)-3(d)). Wild type mice subjected to unpredictable stress exhibit a decreased latency to immobility and an increased total time spent immobile in the forced swim test at 48 hours postpartum compared to unstressed postpartum wild type mice (Table 1; Figures 3(c)-3(d)) (*n* = 5–9 for each experimental group; *∗* denotes *p* < 0.05 using a one-way ANOVA with Tukey's multiple comparisons test; latency: *F*(3,22) = 2.729; total time: *F*(3,22) = 7.874). These data are consistent with the hypothesis that altered stress reactivity plays a role in mediating abnormal postpartum behaviors.

### 3.3. Role of Maternal Corticosterone on Offspring Behavior

**Figure 4:**
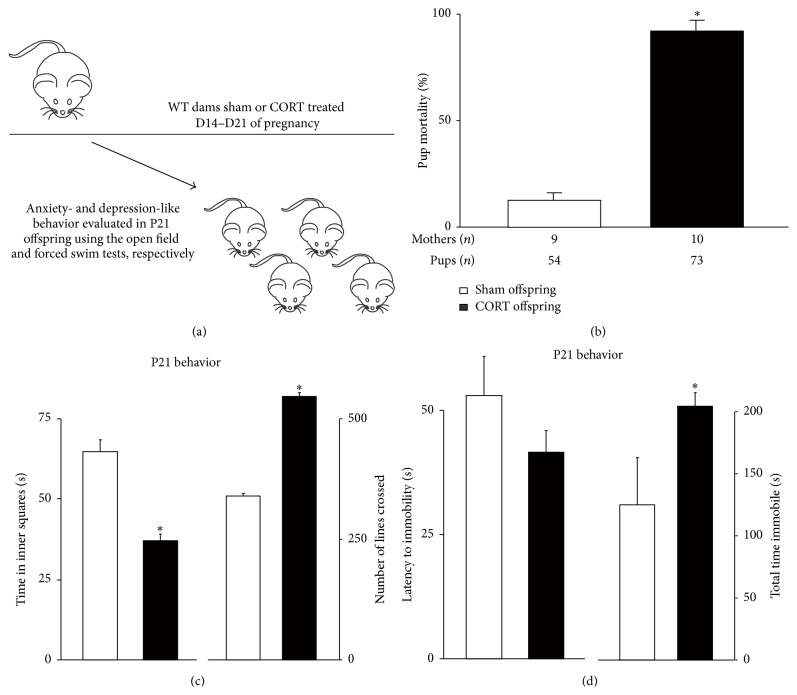
Corticosterone treatment in wild type mice during pregnancy decreases pup survival and induces deficits in offspring behavior. (a) A diagram outlining the experimental design. Wild type dams were implanted with a 10 mg, slow-release corticosterone pellet at D14 of pregnancy and pup survival and offspring behavior in the juveniles were assessed at P21. (b) Corticosterone-treated wild type mice exhibit a decrease in pup survival compared to sham implanted wild type mice. *n*: sham = 9 mothers, 54 pups; CORT: 10 mothers, 73 pups; *∗* denotes *p* < 0.05 using Student's *t*-test. (c-d) Juvenile offspring reared by wild type mothers implanted with a 10 mg, slow-release corticosterone pellet at D14 of pregnancy, exhibit anxiety-like and depression-like behaviors at P21. (c) Juvenile mice reared by corticosterone-treated mothers spent a decreased amount of time in the center of the open field test and an increase in locomotor activity. (d) Juvenile mice reared by corticosterone-treated mothers exhibited a decreased latency to immobility and an increase in the total time spent immobile in the forced swim test (*n* = 8 mice per experimental group; *∗* denotes *p* < 0.05 using Student's *t*-test).

To investigate whether altered stress reactivity in* Gabrd* ^−/−^ mice plays a role in mediating the negative impact of maternal depression-like behaviors on offspring development, we either sham-implanted wild type mice or implanted them with a slow-release 10 mg corticosterone pellet on day 14 of pregnancy and assessed offspring behavior at P21 (Figure 4(a)). We determined that corticosterone treatment does not interfere with pup delivery or litter size (7.3 ± 0.9 pups) compared to sham implanted mice (6.0 ± 0.8 pups) (*n*: sham = 9 mothers, 54 pups; CORT: 10 mothers, 73 pups; *∗* denotes *p* < 0.05 using Student's *t*-test). Note: litter sizes were determined at the time of delivery since there is a decreased survival rate of the pups born to corticosterone implanted mice (Figure 4(b)); however, all pups were alive at the time of delivery. Corticosterone levels are significantly elevated in the corticosterone implanted dams at 48 hours postpartum (192.8 ± 50.3 ng/mL) compared to sham implanted mice (29.8 ± 3.2 ng/mL), postpartum wild type controls (16.8 ± 4.1 ng/mL), or stressed postpartum wild type mice (81.0 ± 10.4 ng/mL) (*n* = 10–15 mice per experimental group; *∗* denotes *p* < 0.05 using Student's *t*-test). Corticosterone treatment in the mothers at D14 was sufficient to induce abnormal postpartum behaviors in wild type mice, such as inability to build a proper nest (data not shown) and an increase in pup mortality rate due to cannibalism or neglect (Table 1; Figure 4(b)), similar to that seen in* Gabrd* ^−/−^ mice [8] (*n*: sham = 9 mothers, 54 pups; CORT: 10 mothers, 73 pups; *∗* denotes *p* < 0.05 using Student's *t*-test). Corticosterone treatment in the mother was also sufficient to induce behavioral deficits in their juvenile offspring. Juvenile mice (P21) reared by mothers treated with corticosterone spent less time in the center squares of the open field test compared to juvenile mice reared by sham implanted wild type mothers (Table 1; Figure 4(c)) (*n* = 8 mice per experimental group, 2 mice per litter in 4 different litters; *∗* denotes *p* < 0.05 using Student's *t*-test), indicative of anxiety-like behavior. In addition, corticosterone treatment alters locomotor behavior in the offspring of corticosterone-treated mothers, evident from the increased number of lines crossed in the open field test compared to sham implanted mothers (Table 1; Figure 4(c)) (*n* = 8 mice per experimental group, 2 mice per litter in 4 different litters; *∗* denotes *p* < 0.05 using Student's *t*-test). Corticosterone treatment in wild type mothers also induced depression-like behavior in the offspring. Offspring reared by corticosterone implanted wild type mothers spend an increased total time immobile during the forced swim test compared to juvenile mice reared by sham implanted wild type mothers (Table 1; Figure 4(d)) (*n* = 8 mice per experimental group, 2 mice per litter in 4 different litters; *∗* denotes *p* < 0.05 using Student's *t*-test). These data support the hypothesis that stress hormones, specifically corticosterone, mediate the negative impact of maternal depression-like behaviors on offspring behavior in the mouse.

**Figure 5:**
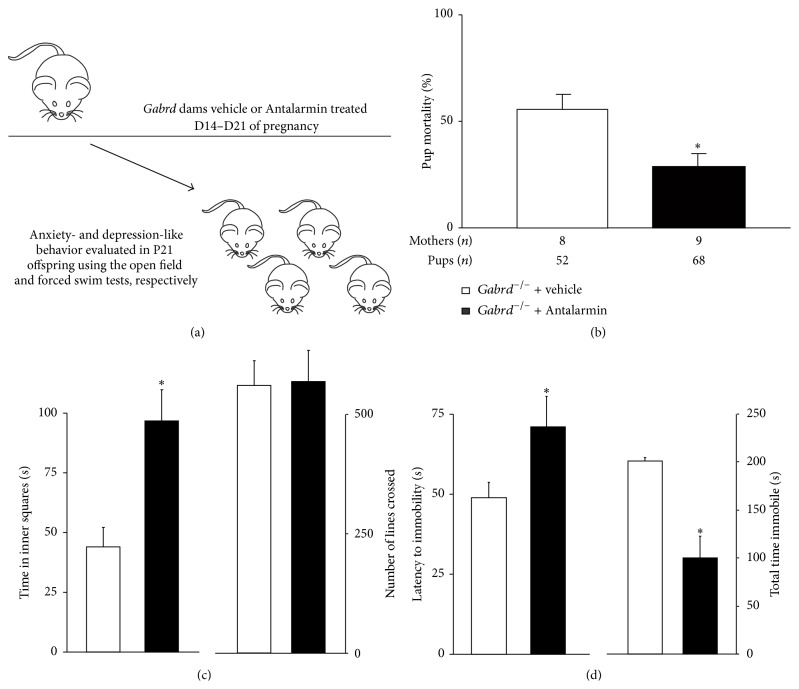
Blocking CRH signaling in* Gabrd* ^−/−^ mice during pregnancy increased pup survival and diminished the negative impact on offspring behavior. (a) A diagram outlining the experimental design.* Gabrd* ^−/−^ dams were treated with Antalarmin from D14 to D21 of pregnancy and pup survival and offspring behavior in the juveniles were assessed at P21. (b) Antalarmin-treated* Gabrd* ^−/−^ mothers exhibit an increase in pup survival compared to vehicle-treated controls. *n*:* Gabrd* ^−/−^ vehicle = 8 mothers, 52 pups;* Gabrd* ^−/−^ + Antalarmin: 9 mothers, 68 pups; significance was determined as *p* < 0.05 using Student's *t*-test. (c-d) Juvenile mice reared by* Gabrd* ^−/−^ mothers treated with Antalarmin exhibit a decrease in anxiety-like and depression-like behavior at P21. (c) Offspring of Antalarmin-treated* Gabrd* ^−/−^ mothers spend an increased amount of time in the center of the open field with no change in activity compared to vehicle-treated controls. (d) Offspring of Antalarmin-treated* Gabrd* ^−/−^ mothers exhibited an increased latency to immobility and a decreased total time spent immobile in the forced swim test (*n* = 8–10 mice per experimental group; *∗* denotes *p* < 0.05 using Student's *t*-test).

If altered stress reactivity in* Gabrd* ^−/−^ mothers plays a role in the impact of maternal depression-like behaviors on offspring development, we hypothesized that inhibiting the stress response in the mother with the corticotropin-releasing hormone (CRH) antagonist, Antalarmin, would decrease the anxiety- and depression-like behaviors in the juvenile offspring (Figure 5(a)). We did not observe any changes in litter size associated with Antalarmin treatment (7.6 ± 0.9 pups) compared to vehicle treatment (6.5 ± 0.8 pups). This dose of Antalarmin was sufficient to decrease the stress-induced circulating corticosterone levels in postpartum* Gabrd* ^−/−^ mice (101.5 ± 12.0 ng/mL) to levels similar to postpartum wild type mice (81.0 ± 10.4 ng/mL) which is significantly lower than the stress-induced levels in postpartum* Gabrd* ^−/−^ mice (384.6 ± 27.6 ng/mL). Antalarmin treatment ameliorated the abnormal postpartum behaviors in postpartum* Gabrd* ^−/−^ mice.* Gabrd* ^−/−^ dams treated with Antalarmin exhibit an increase in pup survival compared to controls (Table 1; Figure 5(b)) (*n*:* Gabrd* ^−/−^ vehicle = 8 mothers, 52 pups;* Gabrd* ^−/−^ + Antalarmin: 9 mothers, 68 pups; significance was determined as *p* < 0.05 using Student's *t*-test). Further, Antalarmin treatment in the mother was also sufficient to ameliorate the mood disorders in juvenile mice reared by* Gabrd* ^−/−^ mothers. Juvenile mice (P21) reared by* Gabrd* ^−/−^ mothers treated with Antalarmin spent more time in the center squares of the open field test, which is indicative of decreased anxiety levels, compared to juvenile mice reared by vehicle-treated* Gabrd* ^−/−^ mothers (Table 1; Figure 5(c)) (*n* = 8–10 offspring per experimental group, 2 mice per litter in 4-5 different litters; significance was determined as *p* < 0.05 using Student's *t*-test). There was no significant difference in the number of lines crossed in the open field test between the offspring of Antalarmin-treated and vehicle-treated* Gabrd* ^−/−^ mothers (Table 1; Figure 5(c)). Similarly, offspring reared by Antalarmin-treated* Gabrd* ^−/−^ mothers exhibit decreased depression-like behavior, evident by an increased latency to immobility and decreased total time spent immobile compared to offspring reared by vehicle-treated* Gabrd* ^−/−^ mothers (Table 1; Figure 5(d)) (*n* = 8–10 mice per experimental group, 2 mice per litter in 4-5 different litters; significance was determined as *p* < 0.05 using Student's *t*-test). These data demonstrate that inhibiting the stress response, such as with the CRH antagonist, Antalarmin, is therapeutic in ameliorating the abnormal postpartum behaviors in* Gabrd* ^−/−^ mothers as well as preventing the negative impact of maternal depression-like behaviors on offspring development.

## 4. Discussion

This study highlights the utility of a unique mouse model to investigate the underlying mechanisms mediating the pathophysiology of postpartum depression and the mechanism(s) through which postpartum depression negatively impacts offspring development. It is generally accepted that both genetic and environmental factors play a role in the pathophysiology of postpartum depression. However, either the current animal models exhibit a genetic predisposition for depression-like behavior or the behavior is environmentally induced [37]. Here we describe a genetic mouse model exhibiting depression-like behavior that is restricted to the postpartum period [8], which is aggravated by environmental stress similar to the human condition [38]. This is the first genetic mouse model which exhibits a predisposition to postpartum depression-like behavior in which there is also an environmental component. Therefore, we feel that this is a useful model for studying the mechanisms mediating postpartum depression-like behavior and the accompanying deficits in offspring development.

Clearly, hormone changes throughout pregnancy and the postpartum period trigger the onset of postpartum depression. However, gonadal hormone levels do not appear to be significantly altered in women with postpartum depression [39–41], suggesting that women must be predisposed to the disorder. During pregnancy, levels of estrogen and progesterone steadily increase due to placental production of these hormones, which decrease abruptly with the removal of the placenta. However, no change in estrogen or progesterone levels has consistently been shown to be associated with postpartum depression [41]. There are numerous other hormonal changes that occur during pregnancy, including changes in oxytocin, prolactin, and cortisol levels (for review see [42]). However, no alterations out of the physiological range were found for prolactin [41, 43], oxytocin, or vasopressin [44] associated with postpartum depression. Hypercortisolism has been suggested to play a role in the pathophysiology of postpartum depression [45], since major depression is also associated with hypercortisolism [46]. Normally, the stress-induced activation of the HPA axis is suppressed during pregnancy ([47], for review see [12]), consistent with our observations in postpartum wild type mice (Figure 2). Altered levels of cortisol [11, 18, 48], ACTH [18], and CRH [19] have been associated with postpartum depression. Researchers have gone so far as to say that elevated CRH levels may be used as a diagnostic criterion for postpartum depression [19]. However, other studies have failed to reproduce these results ([41, 43], for review see [42]). Here we demonstrate hyperresponsivity of the HPA axis associated with depression-like behaviors during the postpartum period in a mouse model, similar to what has been observed in women with postpartum depression [49].

Consistent with a role for HPA axis hyperresponsiveness in postpartum depression-like behaviors, this study supports a role for elevated corticosterone in the pathophysiology of postpartum mood disorders, since physiological stress is sufficient to induce depression-like behavior during the postpartum period in mice (Figure 3). Previous studies have demonstrated that corticosterone alters maternal care and induces postpartum depression-like behaviors in the dams [50]. Here we demonstrate that exogenous corticosterone treatment in wild type mice during pregnancy results in a robust decrease in pup survival (Figure 4). This has previously been observed [51], albeit not to the same extent as in the current study. The discrepancy may be due to a prolonged exposure in our study to levels of corticosterone normally found in stress. However, we cannot rule out potential abnormalities in the pups due to corticosterone exposure which may impact pup mortality. Interestingly, our study demonstrates that physiological stress in the dams is sufficient to increase pup mortality in both wild type and* Gabrd* ^−/−^ mice (Figure 3(b)). However, unpredictable stress does not alter depression-like behaviors in postpartum* Gabrd* ^−/−^ although it increases depression-like behaviors in wild type mice (Figures 3(c) and 3(d)). We interpret these data to indicate that physiological stress is incapable of altering depression-like behaviors in postpartum* Gabrd* ^−/−^ mice in which corticosterone levels are already elevated, demonstrating a potential ceiling effect. Similarly, our results demonstrate that the CRH antagonist, Antalarmin, increases pup survival in* Gabrd* ^−/−^ mice (Figure 5). These data demonstrate a direct role of stress hormones in postpartum mood disorders and may contribute to the negative impact of maternal depression on offspring development.

Children exposed to mothers with postpartum depression exhibit deficits in cognitive development, motor, and emotional development (for review see [52]). Many mechanisms have been proposed to mediate the negative association between maternal depression and offspring development, including environmental and genetic components. It is also possible that there is a direct, biochemical component of maternal depression which impacts offspring development. Deficits in child development associated with maternal depression are correlated with elevated cortisol levels in the mother [53, 54], suggesting that the stress hormones may play a role in the negative impact of maternal depression on child development. Consistent with this theory, we demonstrate behavioral deficits in mice reared by* Gabrd* ^−/−^ mothers, which exhibit hyperresponsiveness of the HPA axis, and offspring reared by wild type mice subjected to US or treated with exogenous corticosterone. Further, corticosterone treatment in dams has previously been shown to result in adverse behavioral effects in the offspring [22], which could be a direct effect of corticosterone levels in the offspring which may alter subsequent HPA axis activity [55]. These data support a direct role of stress hormones in both the pathophysiology of postpartum depression and the negative impact of maternal depression on offspring development.

In the current study, the mouse models which exhibit abnormal postpartum behaviors and a negative impact offspring development, including* Gabrd* ^−/−^ mice and wild type dams subjected to unpredictable stress or treated with exogenous corticosterone, exhibit a high degree of pup mortality due to cannibalism and/or neglect. This is in contrast to the human condition of postpartum depression which is not typically associated with infant mortality or infanticide. Neonaticide and infanticide are more commonly associated with postpartum psychosis [56]. It is possible that postpartum* Gabrd* ^−/−^ mice more accurately model postpartum psychosis. However, this is difficult to assess in mice and requires further study.

Due to the high level of pup mortality in the mouse models exhibiting abnormal postpartum behaviors and a negative impact offspring development, including* Gabrd* ^−/−^ mice and wild type dams subjected to unpredictable stress or treated with exogenous corticosterone, we cannot rule out both the impact of changing litter size on maternal behaviors and the impact on offspring development. Relevant to the current study, smaller litter size is associated with more directed maternal care [57, 58]. Further, mice reared in smaller litters also exhibit decreased anxiety-like behaviors during adulthood [58]. These findings are in contrast to the current study where we observe a decreased litter size in* Gabrd* ^−/−^ litters associated with abnormal maternal care [8] and increased anxiety- and depression-like behaviors in the offspring reared by* Gabrd* ^−/−^ mice (Figure 1). Thus, it does not appear that litter size impacts the findings in the current study.

This study directly demonstrates the negative impact of postpartum depression-like behavior and deficits in maternal care on offspring behavior. Further, our data suggest a role for HPA axis dysfunction in mediating the negative impact of maternal mood on offspring behavior.

## Figures and Tables

**Table 1:** Summary of experimental results.

	Surrogate	Pup survival	Open field		Forced swim test	
		%	Time in center (s)	Beam breaks	Latency to immobility (s)	Total time immobile (s)
Dams						
Wild type		92.0 ± 1.0			70.7 ± 6.2	148.3 ± 26.7
Wild type CUS		56.2 ± 0.9^*∗*^			49.5 ± 8.2^*∗*^	245.6 ± 3.3^*∗*^
*Gabrd* ^−/−^		65.5 ± 0.9^*∗*^			47.7 ± 2.2^*∗*^	205.5 ± 9.8^*∗*^
*Gabrd* ^−/−^ CUS		41.7 ± 1.1^*∗*^			55.8 ± 5.2^*∗*^	222.8 ± 8.1^*∗*^
Wild type sham		88.9 ± 2.7				
Wild type CORT		20.5 ± 4.4^*∗*^				
*Gabrd* ^−/−^ vehicle		51.9 ± 6.2				
*Gabrd* ^−/−^ Antalarmin		75.0 ± 5.1^*∗*^				

Offspring (P21)						
Cross-fostered wild type	WT surrogate		82.9 ± 12.6	324.1 ± 29.1	53.6 ± 2.7	107.1 ± 13.7
Cross-fostered *Gabrd* ^−/−^ mice	WT surrogate		78.6 ± 10.6	331.5 ± 32.4	58.5 ± 6.0	152.9 ± 15.5
Cross-fostered wild type	*Gabrd* ^−/−^ surrogate		43.3 ± 4.3^*∗∗*^	250.5 ± 22.8	39.9 ± 5.3^*∗∗*^	181.4 ± 12.3^*∗∗*^
Cross-fostered *Gabrd* ^−/−^ mice	*Gabrd* ^−/−^ surrogate		40.7 ± 4.2^*∗∗*^	360.4 ± 24.4	40.9 ± 1.4^*∗∗*^	208.4 ± 5.0^*∗∗*^
Wild type sham			65.0 ± 11.2	338.7 ± 20.4	53.0 ± 7.5	124.5 ± 38.2
Wild type CORT			37.0 ± 4.0^*∗∗*^	546.9 ± 18.1^*∗∗*^	41.5 ± 4.5	204.4 ± 11.1^*∗∗*^
*Gabrd* ^−/−^ vehicle			45.1 ± 7.1	560.3 ± 43.9	49.1 ± 4.1	201.0 ± 3.3
*Gabrd* ^−/−^ Antalarmin			96.7 ± 12.8^*∗∗*^	567.0 ± 65.4	71.0 ± 9.3^*∗∗*^	100.0 ± 23.1^*∗∗*^

^*∗*^Significance *p* < 0.05 compared to control, sham, or vehicle.

^*∗∗*^Significance *p* < 0.05 compared to wild type, sham, or vehicle.
